# Peculiarities within peculiarities – dinoflagellates and their mitochondrial genomes

**DOI:** 10.1080/23802359.2017.1307699

**Published:** 2017-04-01

**Authors:** Przemysław Gagat, Dorota Mackiewicz, Paweł Mackiewicz

**Affiliations:** Department of Genomics, Faculty of Biotechnology, University of Wrocław, Wrocław, Poland

**Keywords:** Alveolates, dinoflagellates, genome reduction, mitochondria

## Abstract

After the establishment of an endosymbiotic relationship between a proto-mitochondrion and its probable archaeal host, mitochondrial genomes underwent a spectacular reductive evolution. An interesting pathway was chosen by mitogenomes of unicellular protists called dinoflagellates, which experienced an additional wave of reduction followed by amplification and rearrangement leading to their secondary complexity. The former resulted in a mitogenome consisting of only three protein-coding genes, the latter in their multiple copies being scattered across numerous chromosomes and the evolution of complex processes for their expression. These stunning features raise a question about the future of the dinoflagellate mitochondrial genome.

## 1. Introduction

Mitochondria are two-membrane-bounded cellular ‘powerhouses’ that evolved, very likely, as a result of syntrophy between a *Rickettsia*-like α-proteobacterium and a hydrogen-dependent archaeon around two billions years ago (Martin & Müller 1998; Martin et al. 2015; Wang & Wu 2015; Sousa et al. 2016). Their symbiosis triggered one of the most important transitions in the history of life, namely the transformation of prokaryotes into eukaryotes (Lane & Martin 2010).

During eukaryogenesis, the genome of the proto-mitochondrion underwent a tremendous reductive evolution, involving the loss of several thousand genes, either by being transferred to the host nuclear genome or by becoming irretrievably lost (Martin & Herrmann 1998; Timmis et al. 2004). Highly reduced mitochondria found in e.g. diplomonads, some amoebozoans, and microsporidians are called hydrogenosomes and mitosomes. The latter eliminated all their genetic material, representing an advanced level of reduction (Tachezy 2008; Hjort et al. 2010). The reductive evolution went to extremes in the oxymonad *Monocercomonoides* sp. as it lost the organelle itself (Karnkowska et al. 2016).

**Figure 1.**
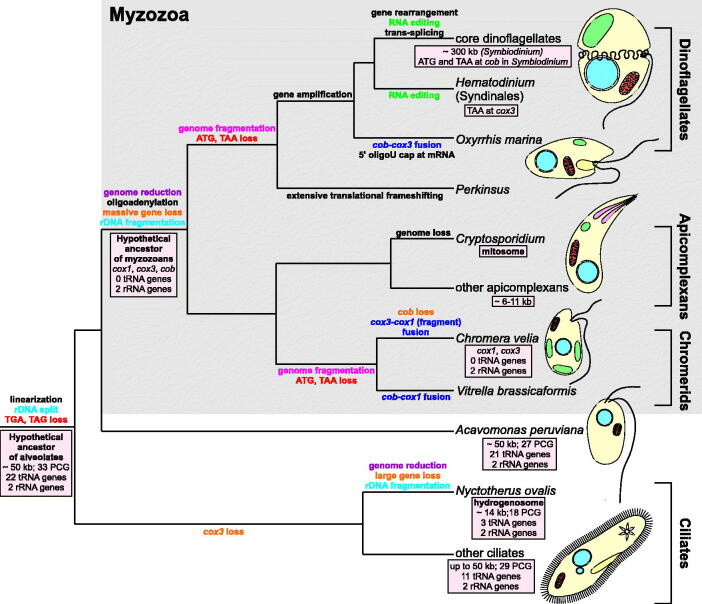
Evolution of mitochondrial genomes in alveolates. The ancestral genome of the superphylum Alveolata was probably about 50 kb in size, such as the mitogenomes of extant ciliates and *Acavomonas peruviana*, and encoded about 60 genes (Janouskovec et al. 2013). Since alveolates diverged about 850 million years ago (Berney & Pawlowski 2006), its mitogenome was subjected to various, often convergent, modifications and transformations as subsequent lineages of the superphylum radiated. Initially, the alveolate mitogenome was linearized from a circular form, lost two stop codons (TGA, TAG) and the genes coding for large and small ribosomal subunits were split into two separately encoded fragments. The most spectacular genome reduction occurred in the common ancestor of myzozoans, i.e. apicomplexans and dinoflagellates, and related lineages (e.g. chromerids), after their divergence from *Acavomonas peruviana* (Janouskovec et al. 2013). Extensive gene loss and gene transfer to the nuclear genome resulted in their extremely small mitogenomes containing only three protein-coding genes (*cox1*, *cox3*, and *cob*), sometimes fused, and two rRNA genes, which were subjected independently to further fragmentation in chromerids and the ancestor of dinoflagellates and perkinsids. This extremely small set of genes was even further reduced in *Chromera velia* as the chromerid completely lost the *cob* gene (Obornik & Lukes 2015). The myzozoan mitogenomes also got rid of about 20 tRNA genes present in the ancestral alveolate genome. At that time, oligoadenylation of transcripts probably evolved. A similar substantial genome reduction also occurred in the anaerobic ciliate *Nyctotherus ovalis*, whose mitochondrion was transformed into a hydrogenosome (de Graaf et al. 2011). This organelle produces hydrogen, which is utilized by methane-producing archaea living together with *Nyctotherus* as endosymbionts in the hindgut of cockroaches (de Graaf et al. 2011). The genome reduction went to extremes in the respiratory and intestinal parasite *Cryptosporidium* that completely lost its mitochondrial genetic material and transformed its mitochondrion into a mitosome, probably involved in Fe-S cluster assembly (Keithly et al. 2005). In dinoflagellates, genes were amplified to numerous copies, which resulted in an increase in their genome size amounting e.g. to ∼300 kb in *Symbiodinium* (Shoguchi et al. 2015). In these protists and sister lineages, various interesting molecular mechanism evolved, such as: translational frameshifting, the addition of 8–9 uridine caps at 5′ end of mRNAs, trans-splicing, and RNA editing (Flegontov & Lukeš 2012). The latter evolved, probably independently, in core dinoflagellates and Syndinales because editing sites are not conserved between these groups, which are separated by lineages without RNA editing (not shown in the figure; Flegontov & Lukeš 2012). It is assumed that universal start (ATG) and stop (TAA) codons, still present in apicomplexans, were independently lost in chromerids and the perkinsid-dinoflagellate branch. Given this model, the presence of TAA at the *cox3* gene in *Hematodinium* (Jackson et al. 2012) as well as ATG and TAA at *cob* in *Symbiodinium* (Shoguchi et al. 2015) implies that these codons may have originated *de novo*. Alternatively, these codons might represent an ancestral state and many alveolates (*Perkinsus*, *Oxyrrhis,* and other dinoflagellates) lost these codons independently. PCG: protein-coding genes.

An interesting evolutionary pathway has been chosen by the mitochondrial genome (mitogenome) of dinoflagellates, which represents a remarkable trend towards simplicity and complexity at the same time. These unicellular protists play an important ecological role as ocean primary producers, parasites, and symbionts, e.g. of reef-building corals (Gómez 2012). They are distinguished by a number of peculiarities, among them the unique mitogenome and, in some cases, even two functional mitochondrial sets in one cell: one of their own and the second present in an engulfed endosymbiotic diatom (Imanian et al. 2012; Gagat et al. 2014). Together with their sister parasitic lineage Apicomplexa and some other relatives, dinoflagellates constitute the Myzozoa assemblage that unites with free-living ciliates in the superphylum Alveolata (Figure 1; Burki 2014).

## 2. Dinoflagellate mitogenome content

The dinoflagellate mitochondrial genome has a similar gene content to the mitogenomes of other myzozoans, which are the most gene-impoverished mitochondrial genomes known (Waller & Jackson 2009; Flegontov & Lukeš 2012). They encode the same set of three divergent protein-coding genes: *cob* (cytochrome b), *cox1* (cytochrome c oxidase subunit 1), and *cox3* (cytochrome c oxidase subunit 3), two highly fragmented rRNA genes for large (LSU) and small (SSU) ribosomal subunits, as well as some uncharacterized small RNA fragments (Shoguchi et al. 2015). Compared to the mitogenome of their closest known Alveolata relative, *Acavomonas peruviana*, the myzozoan mitogenomes must have lost 45 genes (Figure 1; Janouskovec et al. 2013). It is most likely the largest reduction discovered in any aerobic mitochondrion. At least 30 of the 45 genes, including tRNA and NADH dehydrogenase genes, were irretrievably lost. Therefore, myzozoan mitochondria must rely on imported, nucleus-encoded tRNAs to translate their proteins, and on an alternative type 2 NADH dehydrogenase to transfer electrons to ubiquinone (Flegontov et al. 2015). Nine of the 45 genes were transferred to the nuclear genome, e.g. *cox2* (cytochrome c oxidase subunit 2), which is generally present in other eukaryotic mitogenomes. The other six genes, encoding ribosomal proteins, were either lost or their nuclear copies are too divergent to be recognized by computer algorithms (Janouskovec et al. 2013).

## 3. Dinoflagellate mitogenome structure

Although dinoflagellate mitochondrial genomes only have a few genes, they are anything but simple. Pulse field gel electrophoresis experiments indicate that they consist of multiple linear chromosomes with a size of 6–10 kb and longer (see Flegontov & Lukeš 2012 and references therein). The mitogenome size of *Symbiodinium minutum*, a reef-building coral endosymbiont, amounts to ∼326 kb, including mostly (99%) noncoding sequences. It is more than 50 times the size of the *Plasmodium falciparum* mitogenome (an apicomplexan that causes malaria), and 20 times the size of ours (Ji et al. 1996; Shoguchi et al. 2015). Still, the dinoflagellate mitochondrial genomes are smaller than the megabase-sized mitogenomes of some plants, e.g. in the genus *Silene* (*Silene conica* 11.3 Mb; Sloan et al. 2012).

Years of extensive studies have revealed that the large size of the dinoflagellate mitogenome is due to the numerous amplification and recombination events. They resulted in multiple copies of each gene and gene fragments linked in numerous configurations (Nash et al. 2008). The genes are often flanked by species-specific inverted repeats capable of forming stem-loop structures. Although the role of the inverted repeats is unknown, they might be involved, like in other organisms, in mitogenome recombination, replication, and transcript stability (reviewed by Flegontov & Lukeš 2012).

## 4. More peculiarities: missing ORFs boundaries, RNA editing, trans-splicing, and gene fusions

Very few genes and such immense genomes make a unique duet, but there are more peculiarities hidden in the dinoflagellate mitogenomes. First of all, it is difficult to find where the ORFs’ (Open Reading Frames) boundaries are, as the canonical start and stop codons were reported to be missing from the dinoflagellate transcripts (reviewed by Flegontov & Lukeš 2012). However, similar to ciliates and apicomplexans, *S. minutum* does contain unconventional start codons (AUU for Ile, AUA for Ile), and all investigated dinoflagellates use polyadenylation to generate a classical stop codon from an incomplete one ending with uracile in *cox3*, a phenomenon also observed in mitogenomes of some vertebrates. Poly(A) stretches might also constitute a dinoflagellate translation termination signal, e.g. in *cox1*, by causing a kind of ‘sliding’ movement of the ribosome that prematurely terminates translation (Koutmou et al. 2015). Only the *cob* gene, in *S. minutum* at least, has both canonical start and stop codons (Shoguchi et al. 2015). This means that stop codons are still used in dinoflagellates and have not been, for example, reassigned to other amino acids.

The transcripts of all protein-coding genes in dinoflagellates, and some rRNA fragments as well, are edited. The editing intensity increases from basal phylogenetic lineages, where it is absent, e.g. in *Oxyrrhis marina*, to the later branching ones known as the core dinoflagellates (Figure 1; Zhang et al. 2008). Although RNA editing occurs in other organisms, the versatility and scale of changes is unprecedented in dinoflagellates. Nearly all possible substitutions (9 of 12) have been observed and they concern up to 6% of the nucleotides in dinoflagellate transcripts (Waller & Jackson 2009). Generally, editing increases GC content, thereby facilitating the use of nucleus-encoded tRNA. The changes may also eliminate incompatibilities between nucleus- and mitochondrion-encoded proteins, for example, by restoring evolutionarily conserved amino acids (Greiner & Bock 2013). The editing also get rid of an in-frame stop codons, e.g. in *cox1* of *Amphidinium carterae* (Waller & Jackson 2009). Interestingly, beside land plants, dinoflagellates are the only group, for which plastid RNA editing has been reported, however, the process is not as widespread and elaborate as in their mitochondria (Knoop 2011; Smith & Keeling 2015).

The other ‘peculiarity’ concerns *cox3* trans-splicing that is characteristic of all investigated core dinoflagellates (Figure 1; Jackson & Waller 2013). The gene is broken between the regions coding for the sixth and seventh transmembrane helices. Therefore, in order to create the mature *cox3* mRNA, the precursor transcripts must be joined. This process is imperfect because, depending on the species, a certain number of adenosine nucleotides are added between the two mRNAs, e.g. five in *Karlodinium veneficum* and ten in *A. carterae*, which in turn results in one or more lysines in the Cox3 protein. This is, however, tolerated due to the location of the hydrophilic insertion between the transmemebrane domains (Jackson & Waller 2013).

Contrary to split of *cox3* in core dinoflagellates, independent mitochondrial gene fusion has been reported in the early-branching dinoflagellate *Oxyrrhis marina* (*cob*-*cox3*) and the chromerid *Vitrella brassicaformis* (*cob*-*cox1*) (Slamovits et al. 2007; Obornik & Lukes 2015). In *Chromera veila*, a putative *cox3* was also fused with an upstream fragment of *cox1*. As the genes encode subunits of different electron transport complexes, they must function as separate proteins, and therefore they should be (i) translated individually from a polycistronic transcript, (ii) translated from separated transcripts produced by cleavage of pre-mRNA, or (iii) cleaved after their translation. It is also possible that *Oxyrrhis* and chromerids evolved different mechanisms to manage the fused genes and their products.

## 5. Is the dinoflagellate mitogenome going to be lost?

Such a small number of protein-coding genes raises a question about the future of the mitochondrial DNA in dinoflagellates, and other myzozoans as well, especially taking into account the fact that some apicomplexans (*Cryptosporidium* spp.) indeed have highly morphologically and functionally reduced mitochondria without DNA, i.e. mitosomes (Liu et al. 2016). But mitosomes, hydrogenosomes and other mitochondrion-related organelles (reviewed by Makiuchi & Nozaki 2014) have only evolved in parasitic lineages under anaerobic/hypoxic conditions, in which the energetic resources of the host are aplenty. In such environments, the abundance of host metabolites affects not only the mitochondrial genome but also the nuclear and plastid genomes as well. *Cryptosporidium* spp. perfectly exemplifies this through its loss of mitochondrial DNA, the plastid itself, and the great reduction of its nuclear genome in comparison to other eukaryotes, including apicomplexans (reviewed by Liu et al. 2016).

In contrast to *Cryptosporidium* spp., dinoflagellates live in changing, low-nutrient environments that favour highly adaptive and innovative species. In such conditions, organisms are especially under pressure to outcompete rivals, e.g. by growing faster, offering one of reasons why many dinoflagellates are mixotrophs, i.e. can both photosynthesize and feed by phagocytosis (Hansen 2011). The mitogenome loss in dinoflagellates seems highly incomprehensible because it would mean abandoning the most effective way of releasing energy from nutrients, i.e. oxidative phosphorylation. According to the CoRR (Co-location for Redox Regulation) hypothesis (Allen 2015; Allen & Martin 2016), at least one component of each existent respiratory chain complex (complex III and IV in dinoflagellates) must be encoded by the mitogenome to ensure its fine-tuned regulation, and consequently regulation of the entire organelle and cellular metabolism.

It cannot be ruled out that the ancestors of extant myzozoans once led parasitic lives. As a result of a relaxed selection, their mitogenomes were reduced and peculiarities such as, e.g. the missing start/stop codons and gene fragmentation appeared. When dinoflagellates diverged from apicomplexans and changed their trophic strategies, the increased energetic pressure could have triggered their mitogenome inflation. The genome expansion can increase the number of gene copies and consequently their products involved in oxidative phosphorylation, resulting in boosting energy production. Numerous copies also secure mitochondrial genes from accumulating deleterious mutations in an irreversible manner according to Muller’s ratchet (Martin & Herrmann 1998). The other complex processes necessary to express the remaining mitochondrial genes, such as RNA editing, may have evolved, to correct mutations introduced into these genes or increase diversity of their products, like in trypanosomes (Ochsenreiter & Hajduk 2006; Ochsenreiter et al. 2008). All the peculiarities in dinoflagellate mitochondrial genomes observed today result from the reductive forces that have been driving organellar evolution from the very beginning of eukaryogenesis.
